# Identification of an HLA-A*11:01-restricted neoepitope of mutant *PIK3CA* and its specific T cell receptors for cancer immunotherapy targeting hotspot driver mutations

**DOI:** 10.1007/s00262-024-03729-y

**Published:** 2024-06-04

**Authors:** Meiying Shen, Siyin Chen, Xiaojian Han, Yanan Hao, Junfan Wang, Luo Li, Tong Chen, Bozhi Wang, Lin Zou, Tong Zhang, Wanli Zhang, Xiaxia Han, Wang Wang, Haochen Yu, Kang Li, Shengchun Liu, Aishun Jin

**Affiliations:** 1 Department of Breast and Thyroid Surgery, The First Affiliated Hospital of Chongqing Medical University, Chongqing, China; 2 Department of Immunology, College of Basic Medicine, Chongqing Medical University, Chongqing, China; 3 Chongqing Key Laboratory of Basic and Translational Research of Tumor Immunology, Chongqing Medical University, Chongqing, China

**Keywords:** *PIK3CA*, Engineering TCR-T, Neoantigen, HLA-A*11:01, Anti-tumor immunotherapy

## Abstract

Hotspot driver mutations presented by human leukocyte antigens might be recognized by anti-tumor T cells. Based on their advantages of tumor-specificity and immunogenicity, neoantigens derived from hotspot mutations, such as PIK3CA^H1047L^, may serve as emerging targets for cancer immunotherapies. NetMHCpan V4.1 was utilized for predicting neoepitopes of *PIK3CA* hotspot mutation. Using in vitro stimulation, antigen-specific T cells targeting the HLA-A*11:01-restricted *PIK3CA* mutation were isolated from healthy donor-derived peripheral blood mononuclear cells. T cell receptors (TCRs) were cloned using single-cell PCR and sequencing. Their functionality was assessed through T cell activation markers, cytokine production and cytotoxic response to cancer cell lines pulsed with peptides or transduced genes of mutant *PIK3CA*. Immunogenic mutant antigens from *PIK3CA* and their corresponding CD8^+^ T cells were identified. These *PIK3CA* mutation-specific CD8^+^ T cells were subsequently enriched, and their TCRs were isolated. The TCR clones exhibited mutation-specific and HLA-restricted reactivity, demonstrating varying degrees of functional avidity. Identified TCR genes were transferred into CD8^+^ Jurkat cells and primary T cells deficient of endogenous TCRs. TCR-expressing cells demonstrated specific recognition and reactivity against the PIK3CA^H1047L^ peptide presented by HLA-A*11:01-expressing K562 cells. Furthermore, mutation-specific TCR-T cells demonstrated an elevation in cytokine production and profound cytotoxic effects against HLA-A*11:01^+^ malignant cell lines harboring PIK3CA^H1047L^. Our data demonstrate the immunogenicity of an HLA-A*11:01-restricted *PIK3CA* hotspot mutation and its targeting therapeutic potential, together with promising candidates of TCR-T cell therapy.

**Supplementary Information:**

The online version contains supplementary material available at 10.1007/s00262-024-03729-y.

## Introduction

Hotspot driver mutations mostly recur at distinct amino acid sites critical to tumorigenesis [1–3]. These genetic variations are commonly shared by a broad range of cancer types [4]. Notably, neoantigens derived from these hotspot mutations have become center-staged in the realm of targeted cancer immunotherapy [5, 6]. Recent progress in adoptive T cell therapy, particularly with engineered T cells targeting tumor-specific epitopes, has emerged as a cutting-edge strategy against various cancers [7, 8]. The successful use of TCR-T cells in treating solid tumors with the KRAS (G12D) mutation has significantly boosted the rapid expansion of the field of immunotherapy [5]. *PIK3CA*, the gene encoding phosphoinositide 3-kinase alpha (PI3Kα), known for its pivotal role in numerous cellular processes, stands out due to its high mutation frequency in diverse cancer types [4]. Mutations in *PIK3CA*, especially at hotspots like E542K, E545K, H1047R and H1047L, have been correlated with aggressive tumor behavior, recurrence and metastatic spread in endometrial cancer, breast cancer, cervical cancer and colorectal cancer [3, 9–12]. Among these mutations, the histidine to leucine shift at 1047 account for approximately 12% of the cases [13]. Despite the clinical significance of *PIK3CA* neoantigens, there is a conspicuous scarcity in report of their immunogenicity.

Effective TCR-T cell therapy relies on the interaction of TCRs with a cognate neoepitope in the context of an pHLA complex [14, 15]. One of the challenges lie in discerning the neoepitope–HLA combinations, due to the extensive diversity of the HLA alleles and the consequential variation of supertype families [16]. Within the complex HLA system, there are dominantly expressed alleles, such as HLA-A*02:01, HLA-A*24:02 and HLA-A*11:01 [17]. Recent development in mass spectrometry coupled with computational prediction algorithms contributes to the identification of HLA-A*11:01 presented KRAS G12C/D/V neoepitopes, suggesting for the advantages of applying preliminary screening of potential neoantigen-HLA interactions [18, 19]. Another challenge is the laborious cell-based process of TCRs identification, given the vast TCR sequence diversity and the scarcity of neoantigen-specific T cells [20, 21]. Nevertheless, the necessity remains to explore HLA-neoantigen interactions and identify corresponding TCR pools, for promising neoantigen-specific therapeutics in the clinic.

In this study, the immunogenicity of the four most prevalent *PIK3CA* hotspot mutations was characterized by NetMHCpan V4.1. We identified pH1047L, consisted of 9 amino acids (1046–1054) of mutant PIK3CA^H1047L^, could be processed and presented by HLA-A*11:01 molecule. At least two specific TCRs with high functional avidity were verified to successfully recognize and respond to HLA-A*11:01 positive tumor cell lines bearing PIK3CA^H1047L^ mutation. Collectively, our findings provide compelling evidence supporting the notion that HLA-A*11:01 positive cancer patients could be eligible candidates for T cell-based immunotherapies or cancer vaccines targeting the PIK3CA^H1047L^ epitope.

## Materials and methods

### Cell lines

Cell lines K562, Jurkat (Clone: E6-1) and MDA-MB-231 (ATCC) were maintained in RPMI1640 media, supplemented with 10% fetal bovine serum (FBS) (Gibco). SKOV3 (ATCC) was cultured in McCoy’s 5A Medium (Gibco), also supplemented with 10% FBS. Lenti-X 293 T cells (Takara) were propagated in Dulbecco’s Modified Eagle Medium (DMEM) (Gibco), containing 10% FBS, 2 mM GlutaMAX, 1 mM sodium pyruvate (Gibco) and 0.1 mM non-essential amino acids (Gibco). All cultures were maintained in humidified cell incubators containing 5% CO_2_ at 37 °C and regularly tested for mycoplasma contamination.

T cell culture media was comprised of RPMI1640 supplemented with 10% FBS, 1 mM sodium pyruvate, 100 U/mL penicillin, 2 mM GlutaMAX, 55 μM 2-mercaptoethanol and 25 mM HEPES.

RPMI complete media was comprised of RPMI1640 supplemented with 10% FBS, 100 U/mL penicillin, 100 mg/mL streptomycin, 2 mM GlutaMAX and 25 mM HEPES.

Dendritic cell (DC) media was comprised of RPMI complete media supplemented with 800 U/mL GM-CSF and 800 U/mL IL-4.

For generating TRAC/TRBC-dKO-CD8^+^ Jurkat cell line, we introduced the CD8α co-receptor into Jurkat cells, which allows stable the interaction between the TCR and the peptide-major histocompatibility complex (pMHC). We then knocked out the endogenous TCR α-chain (TRAC) and β-chain (TRBC) by CRISPR-Cas9 non-homologous end joining (NHEJ). For generating TAP1-KO-A11^+^ K562 cell line, we knocked out the transporter associated with antigen presentation 1 (TAP1) gene in the K562 cell line by CRISPR-Cas9 NHEJ. Subsequently, we introduced HLA-A*11:01 into cell using the lentiviral system for peptide loading assay. K562, SKOV3 and MDA-MB-231 transduced HLA-A*11:01 using the lentiviral system for co-culture experiments.

## Peptide-HLA binding affinity assay

Peptides were purchase form GenScript at > 85% purity. The lyophilized peptides were diluted to 10 mM in dimethyl sulfoxide (DMSO) and stored at − 20 °C until further use. For the peptide loading assay, TAP1-KO-A11^+^ K562 cells were utilized. These cells were harvested and washed twice with 10 mL of phosphate-buffered saline (PBS). Cells were then resuspended in AIM-V serum-free medium. For the test group, peptides were added to achieve a final concentration of 100 µM, while the negative control group received DMSO only. The cells were incubated at 26 °C overnight. Subsequently, cells were stained with APC-anti-human MHC-I antibody for 15 min at room temperature in the dark. After washing twice with PBS, cells were resuspended and analyzed for HLA expression via flow cytometry. Median fluorescence intensity (MFI) was determined using FlowJo software.

## In vitro induction of mutation-specific T cell

Experiments were performed as reported [22]. Peripheral blood mononuclear cells (PBMCs) were isolated by density-gradient centrifugation using lymphocyte separation medium (Lymphoprep, STEMCELL) and cryopreserved until ready for use.

To generate autologous monocyte-derived dendritic cells (moDCs), the monocytes were enriched from the PBMCs of healthy individuals by using the EasySep CD14 positive selection kit according to the manufacturer’s instructions (STEMCELL). Monocytes were cultured in the DC media. The fresh DC media were added to the cultures at day 3. The moDCs were cryopreserved at day 6 and stored in liquid nitrogen until used in the assays.

Naive CD8^+^ T cells from healthy donors were isolated from PBMC using the naive CD8^+^ T cell isolation kit (STEMCELL) according to the manufacturer’s protocol. The moDCs were pulsed with synthetic peptide at a final concentration of 10 μM and simulated with 10 ng/mL LPS and 100 U/mL IFN-γ into medium for 16 h. Then, naive CD8^+^ T cells were co-cultured with the matured DCs of the same donor for 10 days as described previously [23]. Briefly, cells were seeded in 48-well suspension plates at a density of 5 × 10^5^ per well in T cell culture media supplemented 30 ng/mL IL-21. The ratio of plated CD8^+^ naive T cells to DC cells was 2:1 or 4:1. Culture wells were replenished with a fresh growth medium containing IL-7 and IL-15 (5 ng/ml each) every three days throughout the entire in vitro incubation period.

## ELISPOT assays

IFN-γ ELISPOT assays were performed as reported [24]. Briefly, ELISPOT plates (Millipore) were coated with anti-human IFN-γ antibody (Clone 1-D1K, 2 mg/ml, Mabtech) overnight at 4 °C. T cells were rested in cytokine-free media overnight. Then 2 × 10^5^ T cells were seeded per well in ELISPOT plates and stimulated for 24 h with peptide (10 μM each). Plate-bound 1 μg/mL ΟΚΤ3 was used as the positive control. Subsequently, the plates were incubated with anti-human biotinylated IFN-γ detection antibody (Clone: 7-B6-1, 1 mg/ ml, Mabtech), followed by incubation with Streptavidin-AP (1:1000, Mabtech) and BCIP/NBT-plus substrate (Mabtech). Plates were analyzed using the AID ELISPOT Reader (AID).

## Antibodies and flow cytometry

For surface staining, cells were suspended in FACS buffer and stained with the antibody cocktail for 30 min at room temperature in the dark. The pH1047L-tetramer-PE/APC were used for the identification of antigen-specific CD8^+^ T lymphocytes, which were generated using the PE QuickSwitch Quant HLA-A*11:01 Tetramer Kit (MBL) and APC QuickSwitch Quant HLA-A*11:01 Tetramer Kit (MBL). T cells were stained with the tetramers, anti-CD3 and anti-CD8 antibody cocktails for antigen-specific sorting. T cells were stained with anti-mTCR and anti-CD137 for the activation induced T cell marker assay. Data were acquired via the FACSCelesta cytometer (BD Biosciences, USA) and analyzed using the FlowJo software. The following fluorescently conjugated antibodies were acquired from BioLegend: anti-CD3-PE/BV510 (Clone:SK7), anti-CD4-PerCP-Cy5.5 (Clone:RPA-T4), anti-CD8-FITC (Clone:HIT8a), anti-CD137-BV421/PE (Clone:4B4-1), anti-CD69-PE(Clone:FN50) and anti-mouse TCRβ chain-APC (Clone:H57-597).

## Single-cell sorting and single-cell RT-PCR

T cells were sorted into a 96-well plate. For single T cell PCR, the TCRα and TCRβ chain genes were amplified as previously described [25]. Briefly, single-cell sorted plates were thawed on ice and added with 5 μl mix containing PrimeScript II Reverse Transcriptase (Takara), 2× PrimeSTAR GC Buffer (Takara), RNase-free water, RNase inhibitor, PrimeSTAR HS DNA Polymerase (Takara), dNTP mixture and the primers for TCRα and TCRβ to a total volume of 5 μl. The RT and first PCR program was 45 °C for 45 min, 98 °C for 1 min, 98 °C for 10 s for 30 cycles, 52 °C for 5 s and 72 °C 1 min. The resultant PCR mixtures were diluted 50-fold with water, and 2 μl of the diluted PCR mixtures were added to 18 μl of the nested PCR mixture as the template DNA. The nested PCR program was as follows: 98 °C for 1 min, 98 °C for 10 s for 35cycles, 52 °C for 5 s and 72 °C for 30 s. The PCR products were then analyzed with the *α*-nest or *β*-nest primers by direct sequencing. The TCR repertoire was analyzed with the IMGT/V-Quest tool (http://www.imgt.org/).

## Construction of lentivirus vector and transduction of PBMCs

For the construction of TCR lentivirus vectors, TCRα or TCRβ chains were amplificated from the corresponding nested PCR mixtures and cloned into the lentivirus transfer vector pWPXL (Addgene Plasmid #12257). The constant regions were replaced by mouse counterparts [24]. TCR lentiviruses were generated by co-transfecting Lenti-X 293T cells with the transfer vector and the packaging plasmids psPAX2 (Addgene Plasmid #12260) and pMD2.G (Addgene Plasmid #12259) at a ratio of 5:2:1, using the Xfect Transfection Reagent (Takara). The lentiviral supernatants were harvested after 48 h. PBMCs from healthy donor were stimulated with Dynabeads Human T-Activator CD3/CD28 (Thermo Fisher Scientific) for 24 h and then transduced TCR lentivirus. Fresh culture medium containing 10 ng/ml IL-7/15 was replaced after 8 h.

## CRISPR-Cas9-based method for dual knockout of endogenous TCRɑ and TCRβ chains

A total of 1 × 10^6^ activated T cells were electroporated with CRISPR/Cas9 ribonucleoprotein (RNP) complexes using P3 Primary cell 4D X kit (Lonza:PBP3-02250). We added 5 μL of RNP complexes to T cells in 20 μL of electroporation buffer. Following electroporation, T cells were immediately transferred into a medium containing the lentivirus and incubated for 8 h to facilitate further lentiviral transduction. Every 3 days, cultures were replaced with fresh T cell medium containing IL-7 and IL-15 for a total of 14 days.

## Cytokine secretion assay

The antibody pairs used for cytokine detection are as follows: IFN-γ capture antibody (Clone:MD-1, 2 mg/ml), IFN-γ detection antibody (Clone:4S.B3, 1 mg/ml, Biolegend), IL-2 capture antibody (CloneMQ1-17H12, 4 mg/ml, Biolegend) and IL-2 detection antibody (Clone:Poly5176, 1 mg/ml, Biolegend). The ELISA plate was coated with individual anti-cytokine capture antibodies for 12 h, then washed with phosphate-buffered saline with Tween (PBST) buffer for 3 times, followed by blocking with 3% BSA for at least 1 h at room temperature. Co-culture supernatants of T cell and target cell co-cultures were added into the plate at 50 μl per well. After incubation at room temperature for 1 h, the plate was washed 3 times with PBST. Biotinylated capture antibodies (50 μl/well) were added, and the plate was incubated for 1 h at room temperature, then washed for 5 times. Streptavidin-ALP (1:1000) was added at 50 μl per well and incubated for 1 h at room temperature in the dark. Then, the plate was washed 6 times and the pNPP solution (Mabtech) was added with 50 μl per well. After 20 min incubation at room temperature in the dark, 50 μl stop solution was added per well. The plate was read at 405 nm (OD405) by the Varioskan LUX Multimode Microplate Reader (Thermo Fisher Scientific).

## Bioluminescence-based cytotoxicity assay

In order to establish target cells for bioluminescence-based cytotoxicity assay, a panel of cell lines were generated via lentiviral transduction including MDA-MB-231-luciferase-GFP, MDA-MB-231-HLA-A11-luciferase-GFP, SKOV3-luciferase-GFP and SKOV3-HLA-A11-luciferase-GFP. The capacity of TCR-T cells to lyse tumor cells was quantified employing the OneLumi™ Firefly Luciferase Assay Kit (Beyotime), in accordance with the manufacturer’s protocol. Initially, target cells were seeded at a density of 1 × 10^4^ cells per well in a 96-well plate and incubated overnight to achieve adherence. Subsequently, TCR-T cells, at varying effector-to-target (E:T) ratios (20:1, 10:1, 5:1 and 2.5:1), were introduced to the co-culture and incubated for a period of 24 h. Following incubation, the cell culture plates were placed at room temperature for 10 min, followed by the addition of 100 μl of OneLumi™ detection reagent to each well. It was then incubated at room temperature for 5 min to allow the luminescent signal to stabilize. Luminescence, indicative of cell viability, was quantified as Relative Luminescence Units (RLUs) using the Varioskan LUX Multimode Microplate Reader. Cell viability and cytotoxicity were quantitatively assessed based on luminescence measurements. Luminescence readings from wells devoid of T cells served as the baseline for 100% survival. The survival rate of tumor cells in the presence of TCR-T cells was determined by comparing the luminescence of treated wells to that of the control wells. The cytotoxicity rate was determined as 1 minus the survival rate. Each condition was replicated in triplicate.

## Statistical analysis

All statistical analyses were performed with Graphpad Prism 9.0 (GraphPad). Differences were tested by student’s *t*-test. Error bars represent the SD, and *p* < 0.05 was considered statistically significant. *p < 0.05, **p < 0.01, ***p < 0.001 and ****p < 0.0001 unless otherwise indicated. ns denotes not significant. All data presented are representative of two or more independent experiments.

## Results

### Database analysis of *PIK3CA* high-frequency mutation sites

To identify potential epitopes encompassing known hotspot mutation regions of *PIK3CA*, we employed NetMHCpan V.4.1 for peptide screening. The analysis was restricted to the three most commonly occurring HLA-A alleles (Table 1). NetMHCpan V.4.1 predicted two HLA-A*11:01-restricted peptides with high affinity surrounding PI3Kα E542K and H1047L. The E542K mutation, characterized by a glutamic acid (E) to lysine (K) amino acid exchange, generated a 10-mer peptide epitope (pE542K, sequence: AISTRDPLSK), which was predicted with HLA-A*11:01 binding affinity of 99.32 nM (Table 1). The H1047L mutation gave rise to a 9-mer peptide epitope (p H1047L, sequence: ALHGGWTTK). The binding affinity of pH1047L predicted by NetMHCpan-4.1 was 121.15 nM (Table 1). This analysis provided at least two epitopes derived from *PIK3CA* public mutations with substantially high binding affinity for HLA-A*11:01.

**Table 1:** Summary of *PIK3CA* neoantigens predicted by NetMHCpan V4.1

HLA restriction	*PIK3CA* mutation	Peptide sequence	Peptide length	Position	%Rank_EL	%Rank_BA	Affinity (nM)	Binding level
HLA-A*11:01	PIK3CA_E542K	AISTRDPLSK	10	533–542	0.312	0.566	99.32	Strong
HLA-A*11:01	PIK3CA_E542K	ISTRDPLSK	9	534–542	0.629	1.297	333.21	Weak
HLA-A*11:01	PIK3CA_E542K	STRDPLSK	8	535–542	1.149	3.193	1880.1	Weak
HLA-A*11:01	PIK3CA_E542K	KAISTRDPLSK	11	532–542	0.273	1.107	256.36	Strong
HLA-A*24:02	PIK3CA_E545A	TAQEKDFLW	9	247–252	1.693	3.427	6436.6	Weak
HLA-A*02:01	PIK3CA_H1047L	FMKQMNDAL	9	168–176	0.827	1.004	89.17	Weak
HLA-A*11:01	PIK3CA_H1047L	ALHGGWTTK	9	1046–1054	0.23	0.665	121.15	Strong

Peptide: amino acid sequence of the potential ligand. %Rank: rank of the predicted binding score compared to a set of random natural peptides. Strong binders are defined as having %rank < 0.5, and weak binders with %rank < 2

## HLA-A*11:01-restricted* PIK3CA* mutant epitope is immunogenic and recognized by T cells

**Fig. 1:**
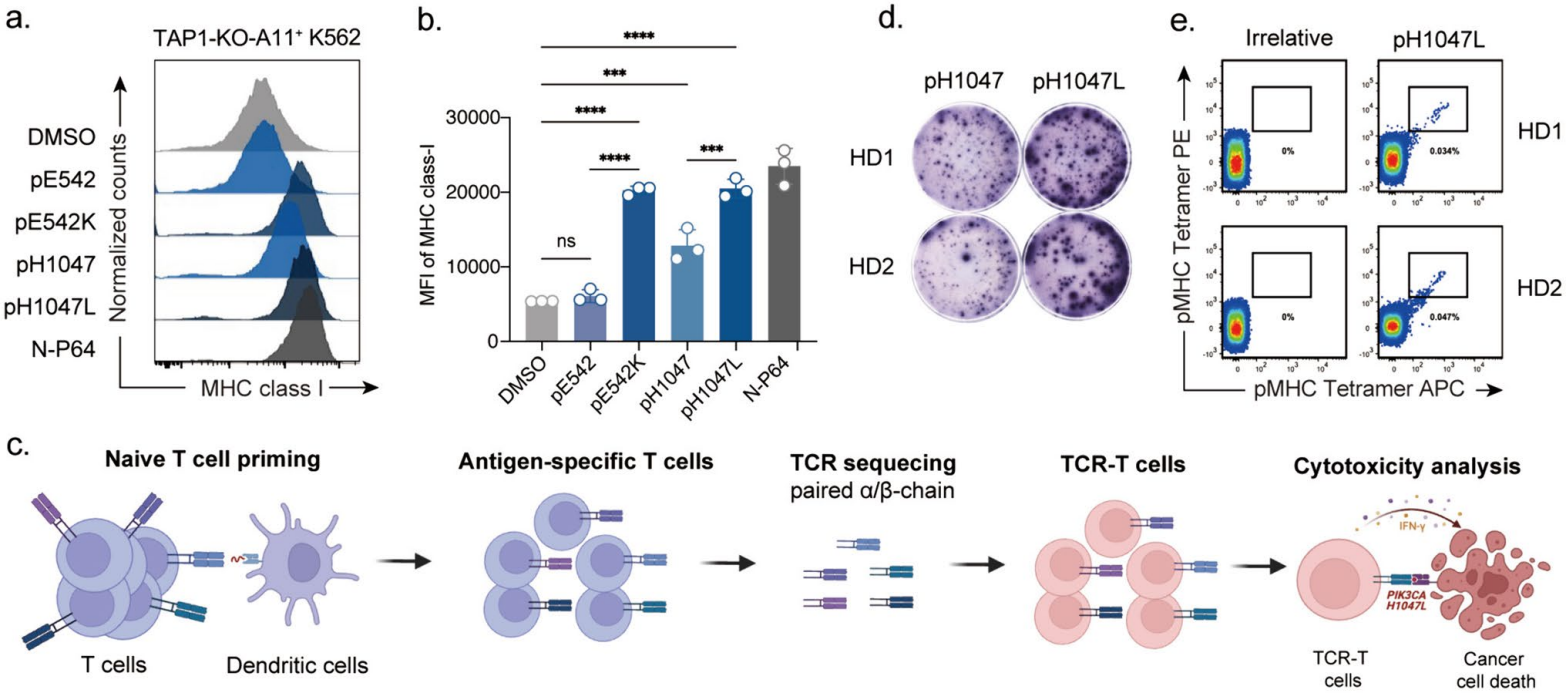
Identification of HLA-A*11:01-restricted T cell epitopes of mutant PI3Kα. **a** Stabilization analysis of HLA-A on TAP1-deficient K562 cells expressing HLA-A*11:01, which **b** median fluorescent intensity (MFI) from three independent experiments. **c** Naïve CD8^+^ T cells were isolated from HLA-A*11:01 positive healthy donors (HDs) and stimulated with pE542K and pH1047L peptides. After 2 rounds of stimulation, the response of T cells was determined by IFN-γ ELISpot after pulsed with wild type and mutant peptides. **d** Tetramer staining showing the percentage of gated tetramer^+^CD8^+^ T cells. **e** Experimental procedure for the isolation of pH1047L-specific T cells from HDs. CD8^+^ T cells were repetitively stimulated with pH1047L-pulsed DCs. pH1047L-specific T cells were sorted by flow cytometric analysis, and selected TCR clones were sequenced. TCR-T cells were engineered and characterized by cytokine production and cytotoxicity. IFN-γ, interferon gamma; ELISpot, enzyme-linked immunospot. N-P64: SARS-CoV-2 N protein 361-369 (KTFPPTEPK). Differences were tested by student’s *t*-test ***: *p* < 0.001; ****: *p* < 0.0001; ns: denotes not significant

To ascertain whether the analyzed *PIK3CA* mutant epitopes could be presented by HLA-A*11:01, we conducted a cell-based peptide loading assays. The HLA-A*11:01^+^ K562 cells with TAP1 deficiency (TAP1-KO-A11^+^ K562) were constructed and pulsed with the predicted 9- or 10-mer *PIK3CA* mutant epitopes and the corresponding wildtype peptides. Flow cytometric analysis showed that the MFI of MHC class I was significantly upregulated with mutant peptides pE542K and pH1047L, to a comparable level to that of a previously verified SARS-CoV-2 epitope capable of forming stable pHLA-A*11:01 complex [24] (Fig. 1a, b). Notably, the wild type peptide pH1047 exhibited binding ability, although to a much lesser degree relative to its mutant counterpart (Fig. 1a, b). These results suggested for the probability of pE542K and pH1047L as prime candidates for eliciting T cell activation.

To assess the immunogenic potential of the *PIK3CA* mutant peptides, we utilized naïve CD8^+^ T cells isolated from HLA-A*11:01 positive healthy donors. These T cells were stimulated in vitro by autologous moDCs pulsed with pE542K and pH1047L, expanded for two rounds and subjected to CD8^+^ T cell reactivity detection evaluation using an IFN-γ ELISPOT assay. The operational workflow of TCR verification is summarized in Fig. 1c. The mutant pH1047L, but not its corresponding wildtype peptide, promoted IFN-γ secretion from the expanded T cell populations (Fig. 1d). However, no pE542 or pE542K responded T cell clones were detected (supplemental Fig. 1a). Furthermore, pH1047L-specific CD8^+^ T cells were detected and sorted by flow cytometry via pH1047L-HLA-A*11:01 tetramer staining (Fig. 1e). A total of five paired TCR *α*- and *β*-chain gene sequences imparting specificity to pH1047L were obtained from these single T cell clones (Table 2). These findings suggested that the predicted *PIK3CA* mutant peptides could establish stable pMHC complex formation, which could elicit active response of CD8^+^ T cells isolated from PBMC of healthy donors.

**Table 2:** Overview of TCR clones identified in this study

TCR ID	Vβ	Vα	CDR3β	CDR3α
TCR1	TRBV5-1*01, TRBD1*01, TRBJ2-1*01	TRAV13-2*01, TRAJ49*01	CASSSGTGGFEQFF	CAEGETGNQFYF
TCR2	TRBV29-1*01, TRBD2*01, TRBJ2-7*01	TRAV12-3*01, TRAJ26*01	CSVSGTSNYEQYF	CATNYGQNFVF
TCR3	TRBV6-1*01, TRBD2*02, TRBJ2-1*01	TRAV24*01, TRAJ28*01	CASSEDFGGSSYNEQFF	CASYSGAGSYQLTF
TCR4	TRBV29-1*01, TRBD2*01, TRBJ2-7*01	TRAV19*01, TRAJ49*01	CSVQTLTSSDEQYF	CALSEATGNQFYF
TCR5	TRBV4-3*01, TRBD2*01, TRBJ2-1*01	TRAV24*01, TRAJ44*01	CASSQDVASSYNEQFF	CASHAGTASKLTF

TCRs were identified following TCRVα and TCRVβ sequencing of flow cytometrically sorted pMHC^+^/CD8^+^ T cells derived from cultures shown in Fig. 1D with CDR3 amino acid sequences specified

## Confirmation of pH1047L-specific TCR expression and functionality in Jurkat cells

**Fig. 2:**
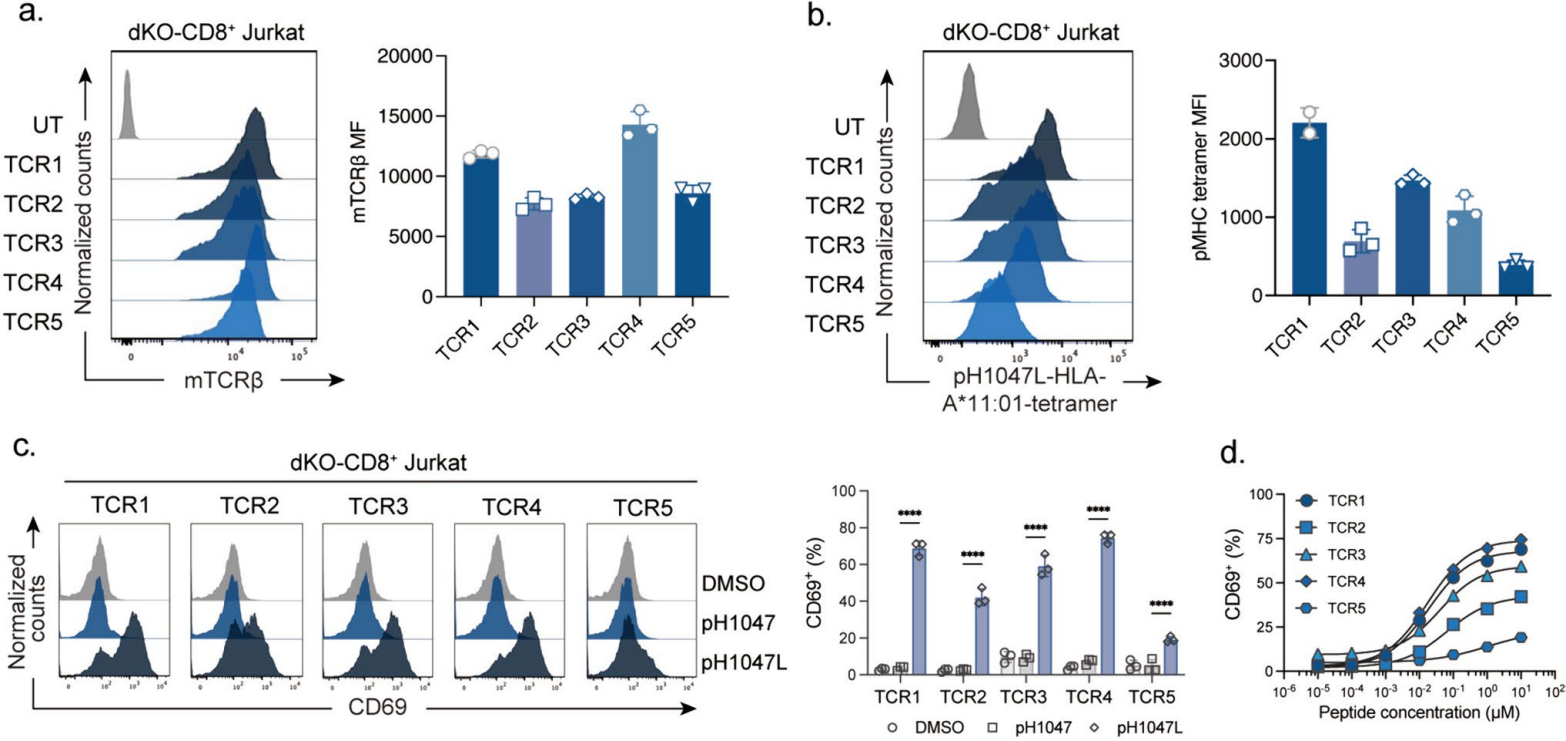
Identification five pH1047L-specific TCR functionality on Jurkat cell. Flow cytometric analysis of **a** mTCRβ expression and **b** pH1047L-HLA-A*11:01 tetramer staining of dKO-CD8^+^ Jurkat cells transduced with pH1047L-specific TCRs (pH1047L-specific TCR Jurkat). **c** The proportion of CD69^+^ cells in pH1047L-TCR Jurkat cells after co-culture with HLA-A*11:01^+^ K562 cells pulsed with or without peptides. **d** The five pH1047L-specific TCR Jurkat cells stimulated for 6 h with HLA-A*11:01-expressing K562 pulsed with titrated peptides. Flow cytometric analysis of CD69^+^ cell proportions in these pH1047L-TCR Jurkat cells. Differences were tested by student’s *t*-test. ****: *p* < 0.0001. mTCRβ: mouse-TCR βchain, UT, untransduced cells

To determine whether the obtained pH1047L-specific TCRs could be successfully expressed and exhibited binding ability to mutant pMHC, we transduced five modified TCRαβ pairs into CD8^+^ Jurkat cells deficient of both *TRAC* and *TRBC* genes (dKO-CD8^+^ Jurkat cells). The levels of TCR expression and their ability to bind to the mutant peptide tetramer were assessed using flow cytometric analysis. Our results showed that all five TCRs manifested on the cellular surface, with TCR1 and TCR4 expressed at advanced levels (Fig. 2a). Furthermore, we found that all five TCRs were able to bind to the pH1047L-specific tetramer, though the tetramer staining intensity varied among them largely (Fig. 2b).

To assess the functional activities of the TCRs, dKO-CD8^+^ Jurkat cells transduced with individual pH1047L-specific TCR were co-cultured with HLA-A*11:01^+^ K562 cells pulsed with 10 μM of either wild type or mutant peptide. We observed significant induction of the proportion of CD69^+^ Jurkat cells exposed to pH1047L relative to the wild type group, suggesting efficient T cell activation induced by all five TCRs (Fig. 2c). Also, the functional avidity of these TCRs was analyzed by addressing the signal of activation, when the TCR-bearing dKO-CD8^+^ Jurkat cells were exposed to titrated concentrations of pH1047L. We found that each TCR exhibited distinct functional avidity, with evident induction of CD69^+^ cell proportions even at a peptide concentration as low as 10 nM (Fig. 2d). Among five TCRs, TCR2 and 5 exhibited relatively weak functional avidity. These data confirmed that all five obtained TCRs could form stable functional CD3-TCR complex, which could specifically bind to pMHC and efficiently promoted TCR downstream signaling activation.

## Functional activation of pH1047L-specific TCR-T cells

**Fig. 3:**
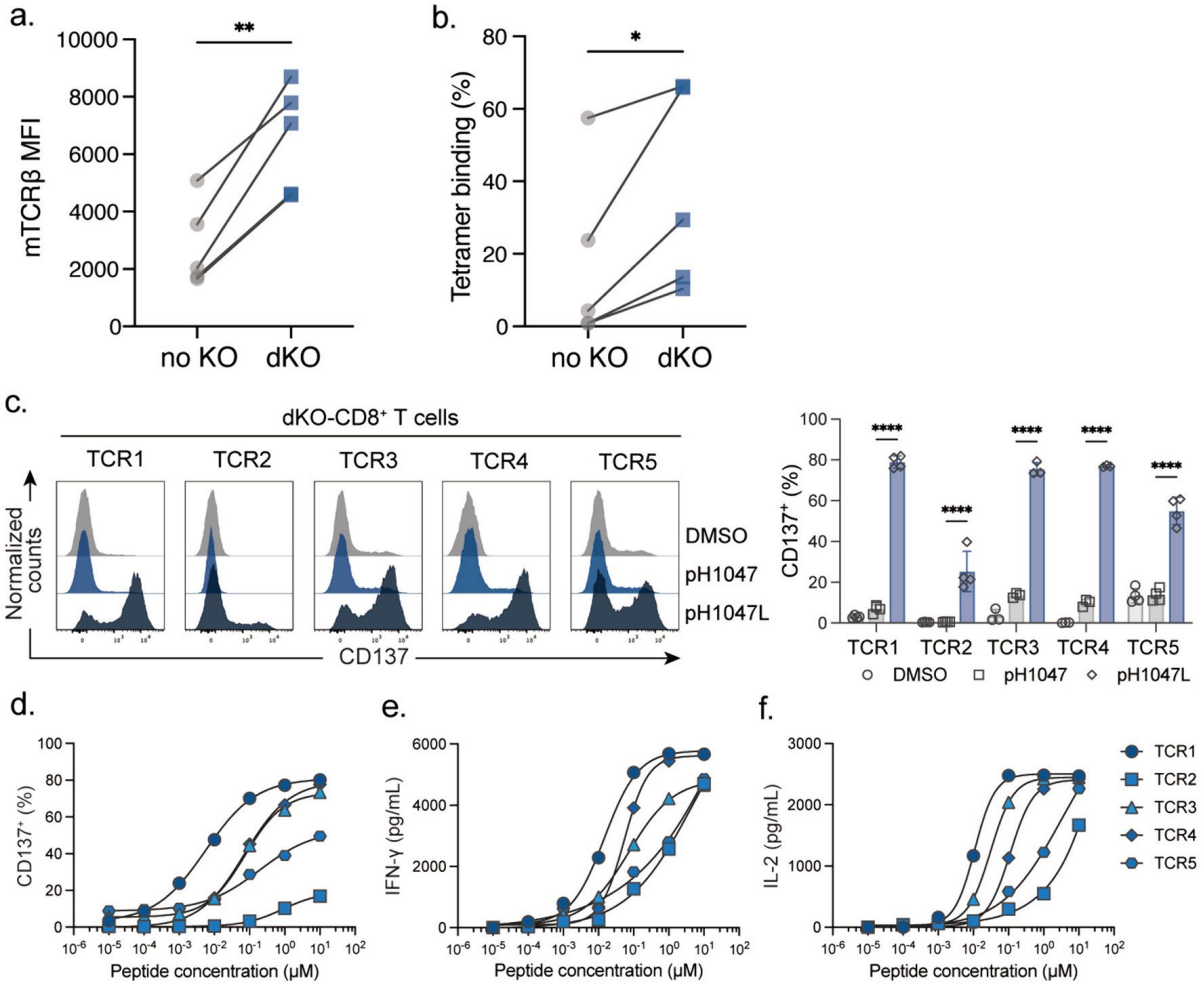
Validation of pH1047L-specific TCR functionality in primary T cells. Primary CD8^+^ T cells were transfected on day 2 post-stimulation with *TRAC/TRBC* knockout (dKO) or not (no KO). Direct comparison of **a** mTCRβ expression and **b** tetramer binding rate between primary CD8^+^ T cells dKO with no KO. Flow cytometric analysis of **c** the expression and **d** the percentage of CD137^+^ pH1047L-specific TCR-T cells after co-cultured with HLA-A*11:01^+^ K562 cells pulsed with wild type or mutant peptide. DMSO was used as control. **e** Flow cytometric analysis of dose-dependency of CD137^+^ cell proportions, pulsed with titrated pH1047L peptide. ELISA of **f** IFN-γ and **g** IL-2 release from pH1047L-specific TCR-T cells. Differences were tested by student’s t-test. *: *p* < 0.05; **: *p* < 0.01; ****: *p* < 0.0001

Further, we engaged primary CD8^+^ T cells isolated from healthy donors that were transduced with individual TCR to assess the functionality of pH1047L-specific TCR-T cells. In order to minimize the undesired effect of endogenous TCRs, we employed the CRISPR/Cas9 system to knock out *TRAC* and *TRBC* genes in these T cells prior to introduction of pH1047L-specific TCRs. The results showed that abrogation of the endogenous TCR *α*- and *β*-chains significantly enhanced the surface expression of all five pH1047L-specific TCRs, and markedly increased their specific binding to the pH1047L-HLA-A*11:01 tetramers (Fig. 3a, b). These results in primary CD8^+^ T cells suggested that mitigating TCRαβ mispairing could efficiently enhance pH1047L-specific TCR cell surface expression, leading to enhanced pH1047L-tetramer binding. When these TCR-bearing CD8^+^ T cells were separately co-cultured with HLA-A*11:01^+^ K562 cells pulsed with pH1047L or wild type peptides, we detected robust induction of CD137^+^ T cell proportions in all five TCR-T groups exposed to pH1047L, which was not seen when in exposure to wild type peptides (Fig. 3c, d). Peptide titration evidenced that TCR1, TCR3 and TCR4 exhibited markedly high functional avidity, capable of elevating CD137^+^ TCR-T cell proportion at nanomolar concentrations (Fig. 3e). In consistence, TCR1, TCR3 and TCR4 exhibited advanced proficiency in IFN-γ and IL-2 production (Fig. 3f, g). These results suggested that pH1047L-specific TCRs could specifically recognize mutant peptide and exhibited HLA restriction, showed high function avidity in T cell activation and cytokine secretion.

## Modeling of TCR–pMHC structure

To further investigate the interaction of TCR–pMHC, 3D models of the five TCR–pMHC complexes were made using TCRmodel2 (http://tcrmodel.ibbr.umd.edu/) [26]. The structural model of pH1047L-specific TCRs in complex with pHLA is depicted in Supplemental Fig. 2. The variable regions TCR Vα and TCR Vβ interact with the pMHC surface and are important during TCR–pMHC recognition [27]. We predicted amino acids of complementarity-determining regions (CDR) α and β loops from pH1047L-specifc TCRs that interact with the pH1047L peptide, labeled the hydrogen bond formed between TCR and mutant peptide. The residues P6-Trp and P8-Thr formed hydrogen bonds with the majority of TCRs, indicating their essential role in the TCR–pMHC interaction (supplemental Fig. 2a, e). To be noted, none of these TCRs displayed cross-reactivity to the closely related HLA-A*03:01, despite the reported presentation of pH1047L by this HLA molecule (supplemental Fig. 3). Structural modeling indicated a similar conformation for pH1047L when presented by HLA-A03:01 and HLA-A11:01, as seen with TCR1 interaction with these two pHLA complexes (Supplemental Fig. 2c). To further analyze TCR–pMHC structure model, it was observed that certain residues in the variable region of TCR1, particularly in CDR1α and CDR2α, might interact with the amino acids located at positions 161 and 163 of HLA-A*11:01. These interactions differ from those observed with HLA-A03:01 (Supplemental Fig. 2d). Nevertheless, rest of the TCRs binds to the relatively conserved region sequences of the two HLAs. The conformation of pH1047L presented by two distinct HLAs showed subtle changed when interaction with TCR2 and TCR4 (Supplemental Fig. 2f, h). These results suggested that the HLA restriction of TCR1 arises from the differing residues between the two HLAs, and TCR2 and TCR4 may result from change of the pMHC conformation.

Further, to investigate how these TCRs distinguish wild type and mutant peptide, we performed structural modeling for pMHC complexes of HLA-A*11:01 presenting wild type or its mutant peptide. The results revealed that they adopt nearly identical conformations when bond with HLA-A*11:01 (supplemental Fig. 4a). The overall similarities of the two complexes suggest that structural differences alone likely cannot explain the TCRs distinguish wild type and mutant peptide. The second residue from the N-terminal of the peptide, embedded in the peptide-binding groove of HLA-A*11:01, was unlikely to directly interact with the TCRs (supplemental Fig. 2b). Combined with the results from the TAP1 deficiency cell-based pMHC stabilization analysis assays (Fig. 1a), we conclude that the specific recognition by pH1047-specific TCR leads to the formation of a stable pHLA complex, rather than directly interacting with mutant residue of the peptide.

## pH1047L-specific TCR-T cells effectively recognized and killed HLA-A*11:01^+^+ PIK3CA^H1047L^ tumor cells

**Fig. 4:**
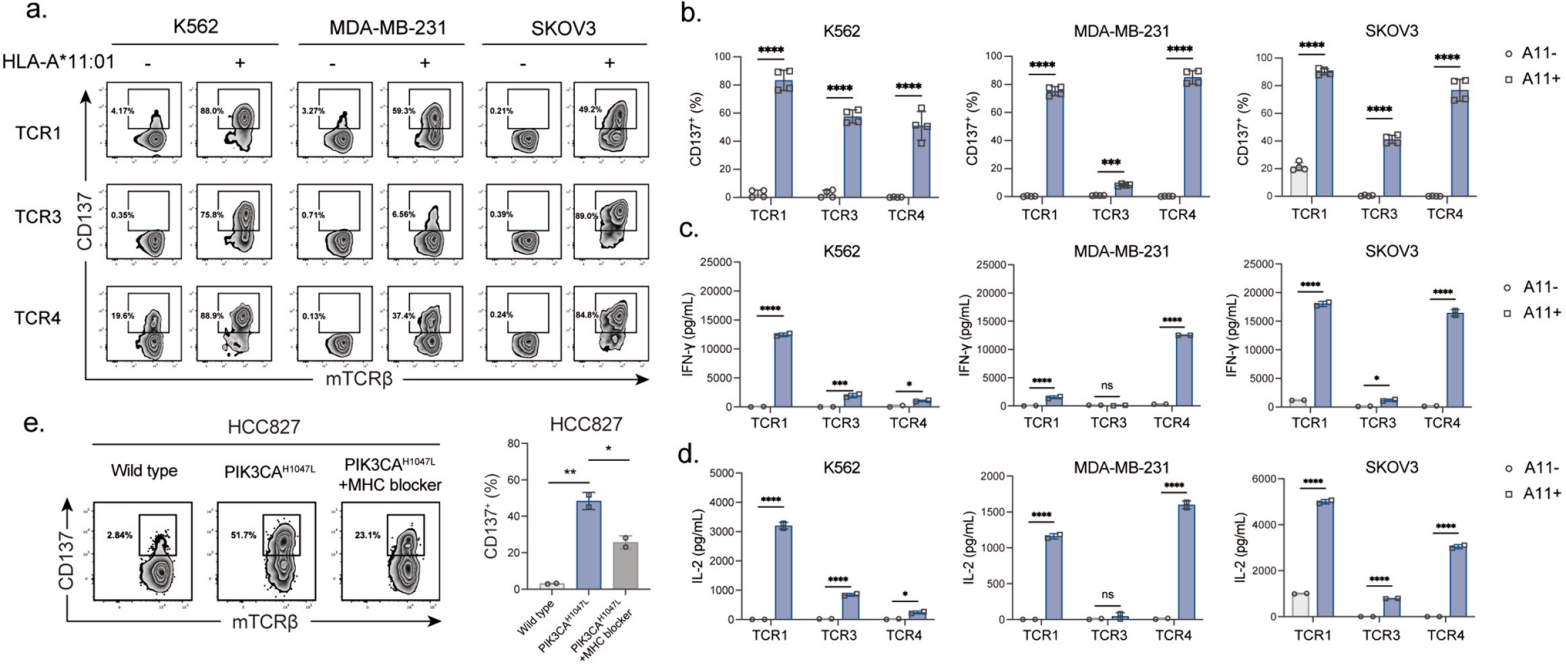
pH1047L-specific TCR-T cells recognize endogenously present PIK3CA^H1047L^. (**a**, **b**). Flow cytometric analysis of the percentage of CD137^+^ dKO-CD8^+^ T cells following overnight co-culture with K562, MDA-MB-231 and SKOV3 cells expressing PIK3CA^H1047L^ at an effector-to-target (E:T) ratio of 1:1. ELISA of **c** IFN-γ and **d** IL-2 release from activated pH1047L-specific TCR-T cells. **e** The pH1047L-specific TCR1-T cells were co-cultured with HCC827 cancer cells endogenously expressing HLA-A*11:01 overnight. The percentage of CD137^+^ TCR-T cells was analyzed via flow cytometry. An MHC blocker (clone W6/32) was added to block the TCR–pMHC interaction. Differences were tested by student’s *t*-test. *: *p* < 0.05; **: *p* < 0.01; ***: *p* < 0.001; ****: *p* < 0.0001

To verify whether pH1047L could be endogenously presented by HLA-A*11:01 positive target cells, we examined the TCR-T cell, generated from primary CD8^+^ T cells, response to various cancer cell lines expressing PIK3CA^H1047L^ and HLA-A*11:01. Flow cytometric analysis demonstrated that the proportions of CD137^+^ TCR-T cells were significantly elevated, when TCR1-, TCR3- and TCR4-T cells were co-cultured with HLA-A*11:01 transduced K562, MDA-MB-231 or SKOV3, relative to their corresponding cell lines without ectopic expression of HLA-A*11:01 (Fig. 4a, b). In parallel, we assessed IFN-γ and IL-2 production from stimulated pH1047L-specific TCR-T cells. All three TCRs responded to PIK3CA^H1047L^-bearing HLA-A*11:01 positive tumor cell lines, whereas the lack of HLA-A*11:01 failed to demonstrate anti-tumor reactivity (Fig. 4c, d). Comparatively, the activation levels of TCR1- and TCR4-T cells were more profound than that of TCR3-T cells (Fig. 4b, c, d). Additionally, a significantly elevated proportion of CD137^+^ TCR1-T cells was detected when they were co-cultured with PIK3CA^H1047L^-transduced HCC827 cells, which was a cell line naturally expressing HLA-A*11:01 (Fig. 4e). This activation of TCR1-T cells was partially abrogated in presence of an MHC blocker (Fig. 4e). These results confirmed that pH1047L could be processed and presented by HLA-A*11:01 positive tumor cells, which could be recognized by pH1047L-specific TCR-T cells.

**Fig. 5:**
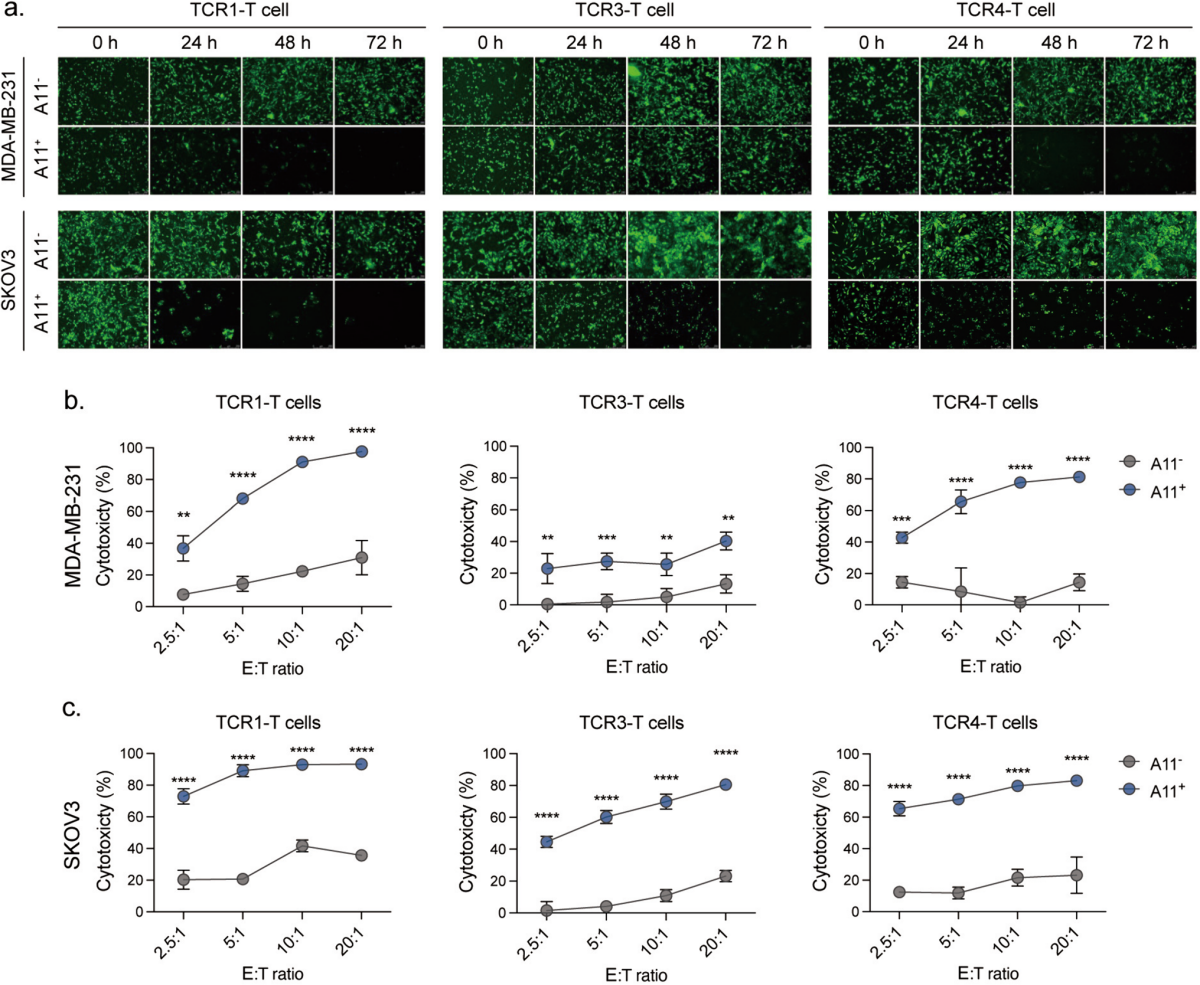
pH1047L-specific T cells specifically recognized and killed HLA-A*11:01^+^ PIK3CA^H1047L^-bearing tumor cells in vitro**. a** pH1047L-specific T cells were co-cultured with PIK3CA^H1047L^-bearing A11^−^ or A11^+^ MDA-MB-231 and SKOV3 cancer cells at an E:T ratio of 20:1 for 0, 24, 48 and 72 h. The fluorescence microscopy images of cytotoxicity are shown. Scale bar, 250 μm. (**b**, **c**) Cytotoxicity of TCR-T cells was assessed by luciferase assays upon 24 h co-culturing with PIK3CA^H1047L^-bearing A11^−^ or A11^+^ MDA-MB-231 and SKOV3 cells labeled with luciferase. Target cell survival was assessed by luciferase at each tested E:T ratio. Differences were determined by student’s *t*-test. **: *p* < 0.01; ***: *p* < 0.001; **** *p* < 0.0001

Further, the kinetics of cell lysis was studied by examining the fluorescence of EGFP-labeled tumor cells. The number of viable tumor cells was apparently reduced over a course of 72 h, suggesting sustained cytotoxicity from these TCR-T cells activated by endogenously presented pH1047L in the HLA-A*11:01 positive target cells (Fig. 5a). Moreover, the cytotoxicity assay showed specific lysis of both HLA-A*11:01 positive MDA-MB-231 and SKOV3 cells induced by TCR1-, TCR3- and TCR4-transduced T cells, and the cytotoxic levels increased in an E:T ratio dependent manner (Fig. 5b, c). Taken together, tumor cells could process and present the PIK3CA^H1047L^ neoantigen, which exhibits HLA-A*11:01-restricted immunogenicity to CD8^+^ T cells. The identified pH1047L-specific TCRs are potentially applicable for TCR-T cell therapy targeting PIK3CA^H1047L^-bearing tumors.

## Discussion

Adoptive transfer of genetically engineered T cells has become one of the most promising avenues of cancer therapy, with commanded selectivity for mutated epitopes and a low risk of off-tumor toxicity [5, 28]. Recent findings evidenced the possibility of TCR-T cell immunotherapy to directly target cancer neoantigens via the pHLA pathway [29]. Here, we provided PIK3CA^H1047L^, an applicable hotspot driver mutation with HLA-A*11:01 restriction. We utilized an optimized comprehensive screening regimen that allowed for the efficient identification of tumor-derived mutant antigens and their corresponding TCRs. The obtained pH1047L, consisted of 9 amino acids (1046–1054) of mutant PIK3CA^H1047L^, successfully provoked immune responses from T cells isolated from healthy donors. Stratification of corresponding TCR panel revealed that this neoepitope could induced T cells with high function avidity and capable of proficient cytolysis of PIK3CA^H1047L^-bearing HLA-A*11:01 positive target cancer cells. Our findings embarked on the pHLA-specific therapeutics by presenting a neoantigen for the most common genetically altered driver genes with immunogenicity, in the context of a prevalent HLA-I allele.

*PIK3CA* is mutated in approximately 30–40% of all human cancers. Predominantly, mutations encompass alterations at E542K, E545K, H1047R and H1047L loci contribute to 80–90% of observed *PIK3CA* genetic disruptions [4, 13]. These specific mutations are confirmed oncogenic drivers, particularly in hormone receptor-positive breast cancers that are often associated with recurrence and metastasis [30]. In addition to a crucial role in cancer progression, a paucity of discovered *PIK3CA* neoantigens has been noticed [31, 32]. Among the top clinically associated *PIK3CA* hotspot mutations, only the PIK3CA^H1047L^ neoantigen was identified with the prevalent HLA-I alleles in our systematic investigation. HLA-A*11:01 has been reported to be one of the most prevalent class I HLA allele in Chinese, with frequencies up to 40%, and approximately 23% in Asian-Americans [33]. Identifying the novel pair of pH1047L-HLA-A*11:01 expanded the known immunotherapeutic target pool, specifically for tumors harboring the *PIK3CA* mutation.

To date, studies regarding the HLA restriction of *PIK3CA* hotspot driver mutations are limited. In line with a previous finding, the HLA-A*11:01-restricted PIK3CA^H1047L^ mutation epitope was shown to be concurrently presented by HLA-A*03:01 [32]. The fact that this neoepitope could be presented by both HLA molecules might largely be consequential to the high degree of structural similarity between their peptide-binding grooves, leading them to share overlapping peptide presentation repertoires [34]. Both of these HLA belong to A3 supertype family, and cross-presented peptides possess similar features such as preferences of Ala, Leu, Ile, Val, Met, Ser or Thr at position 2, and positively charged Arg or Lys at the C-terminus of the peptides [35]. Our data revealed that the P2 amino acid changes from H to L amino acid in enhancing the stability of pMHC across both HLA subtypes (Fig. 1a, supplemental Fig. 1A). Mutations, particularly those at anchor positions, enhance peptide-MHC binding, leading to improved epitope presentation. This presentation, to which the immune system has not been tolerized, offers crucial insights for tumor vaccine development.

The intricate dynamics of T cell recognition in relation to HLA molecules have been a subject of considerable interest in immunological research. Previous study showed that those HLA belong to same supertype family alleles shared partial cross-recognizing T cells in a few individuals [36]. Nonetheless, we observed that TCRs recognizing the PIK3CA^H1047L^ neoantigen presented by HLA-A*11:01 conspicuously lacked cross-reactivity with that presented by HLA-A*03:01. This raises pertinent questions about the underlying factors that influence TCR recognition specificity. Despite the high similarity between these two molecules, they possess polymorphisms in seven amino acids, all situated within the 1 (G-ALPHA1) and 2 (G-ALPHA2) domains, potentially affecting peptide binding and TCR recognition [37]. Three of these amino acids are located in the peptide-binding groove, potentially contributing to subtle variations in peptide presentation. We speculated that the HLA restriction of the five pH1047L-specific TCR recognition arises from the polymorphic residues within the regions of the HLA alleles or distinct conformations of pMHC. More details still need to be verified by analysis of the crystal structure.

While significant efforts have been made to enhance TCR affinity for cancer-associated antigens in ACT, substantial increases in this affinity can lead to heightened cross-reactivity, potentially causing adverse clinical events [38]. An in-depth analysis of TCR sequences and a comprehensive structural examination of TCR–pMHC interactions are foundational for both the advancement of TCR-T cell therapies and the broader understanding of immune system function [39, 40]. In our study, we identified a total of five antigen-specific TCRs and conducted an in-depth analysis of their VDJ gene subtypes. The TRAV and TRBV segments comprising these TCRs were derived from distinct allelic genes, showing no discernible subtype bias. Recent research has suggested that antigen-specific TCR repertoires can exhibit motif features in the CDR3 region of the beta chain based on TCR clustering analysis algorithms such as GLIPH [41]. While our set of five TCRs did not exhibit these characteristic motif features. Moreover, by employing TCR prediction models, we could systematically analyze TCR–pMHC interactions for those identified TCRs, predicted the molecular determinants governing TCR specificity and sensitivity.

Sufficient expression of exogenous TCRs exhibits paramount role for the efficacy of TCR-based immunotherapy [42]. Contemporary study has evidenced the impediment of successful expression and functionality of introduced TCRs by endogenous TCRs in engineered T cells [43–45]. Therefore, we expected an increased efficiency in epitope recognition and cytolytic reaction for TCR-T cells with targeted knockout of endogenous TCRαβ chains. Indeed, the implemented optimization of TCR-T cell engineering enhanced the efficiency of pMHC tetramer binding. Our results supported the concept of endogenous TCR removal during TCR-T cell production process, which would serve as a substantial improvement for clinical applications of adoptive therapy.

Another significant aspect for the identified *PIK3CA* mutant neoantigen and the corresponding TCRs resides within the applicable platform of tumor vaccine. Targeting hotspot mutations, like PIK3CA^H1047L^, can largely encompass precise tumor specificity and dominance. This validated epitope is expected to induce tailored immune responses, consistent with ongoing efforts via similar strategies designing personalized melanoma vaccines by various research groups. Moreover, these functional TCRs could serve as a catalyst in augmenting the spectrum of TCR-T cell therapeutic candidates or soluble bispecific TCRs, aiming for pan-cancer tumors with HLA-A*11:01 restriction. Notably, Kimmtrak stands as the world’s first approved TCR therapy, further indicating the great potential of TCR-T therapy [46]. While our findings are promising, further in vivo studies and clinical trials are warranted to ascertain the efficacy and safety profiles of the identified target and therapeutic modalities.

In conclusion, our research contributed a novel combination of neoantigen and HLA, with a specific focus on TCRs targeting neoepitopes of the oncogenic PIK3CA^H1047L^ mutation. Our findings offer valuable insights into the validation of potentially actionable neoepitope targets for immune-based therapies.

### Supplementary Information

Below is the link to the electronic supplementary material.

Supplementary Figure 1. (A) The response of T cells was determined by IFN-γ ELISpot after pulsed with wild type and mutant peptide. (TIF 2114 KB)

Supplementary Figure 2. (A) Structural overview of the TCR1 pH1047L/HLA-A*11:01 ternary complex. Yellow TCRα chain, orange TCRβ chain, green pH1047L, violent HLA-A*11:01. (B) AAs of TCR1’s CDR3α and CDR3β loops that interact with the pH1047L peptide. Hydrogen bonds are indicated by red. (C) Top view of the conformation of pH1047L peptide presented by HLA-A*11:01 or HLA-A*03:01, when interaction with TCR1. Pink HLA-A*03:01, blue HLA-A*11:01. (D) Top view of the pH1047L/HLA-A*11:01 complex displaying the positions of the six CDR loops of TCR1. Blue AA indicate the different amino acid between HLA-A*03:01 and HLA-A*11:01. (E) Structural overview of the TCR2, 3, 4 and 5 pH1047L/HLA-A*11:01 ternary complex. (F) AAs of TCR2, 3, 4 and 5’s CDR3α and CDR3β loops that interact with the pH1047L peptide. (G) Top view of the pH1047L/HLA-A*11:01 complex displaying the positions of the six CDR loops of TCR2, 3, 4 and 5. (H) Top view of the conformation of pH1047L peptide presented by HLA-A*11:01 or HLA-A*03:01, when interaction with TCR2, 3, 4 and 5. Pink HLA-A*03:01, blue HLA-A*11:01. (TIF 32148 KB)

Supplementary Figure 3. (A) Flow cytometric analysis of the percentage of CD137^+^ pH1047L-specific TCR-T cells after co-cultured with HLA-A*03:01^+^ K562 cells pulsed with mutant peptide. DMSO was used as control. (TIF 3214 KB)

Supplementary Figure 4. (A) Structural superimposition of the pH1047L and pH1047 peptides bound to HLA-A*03:01 or HLA-A*11:01. (B) Stabilization analysis of HLA-A on TAP1-deficient K562 cells expressing HLA-A*03:01. Differences were tested by student’s *t*-test. **: p < 0.01. ns: denotes not significant. (TIF 8083 KB)

## Data Availability

The datasets generated during and/or analyzed during the current study are available from the corresponding author on reasonable request.
