# Supplementary figures and images for: Identification of an HLA-A*11:01-restricted neoepitope of mutant PIK3CA and its specific T cell receptors for cancer immunotherapy targeting hotspot driver mutations
Source: Cancer Immunol Immunother. 2024 Jun 4;73(8):150. doi: 10.1007/s00262-024-03729-y (PMC11150344; doi:10.1007/s00262-024-03729-y)

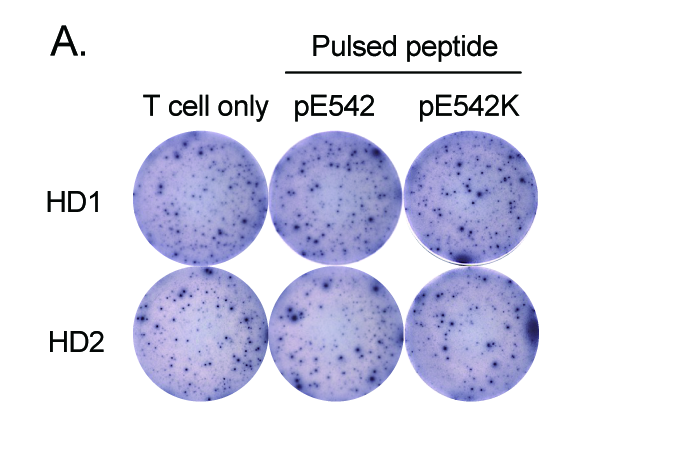

Supplement: Supplementary file 1 — Supplementary Figure 1. (A) The response of T cells was determined by IFN-γ ELISpot after pulsed with wild type and mutant peptide. (TIF 2114 KB) [file 262_2024_3729_MOESM1_ESM.tif]

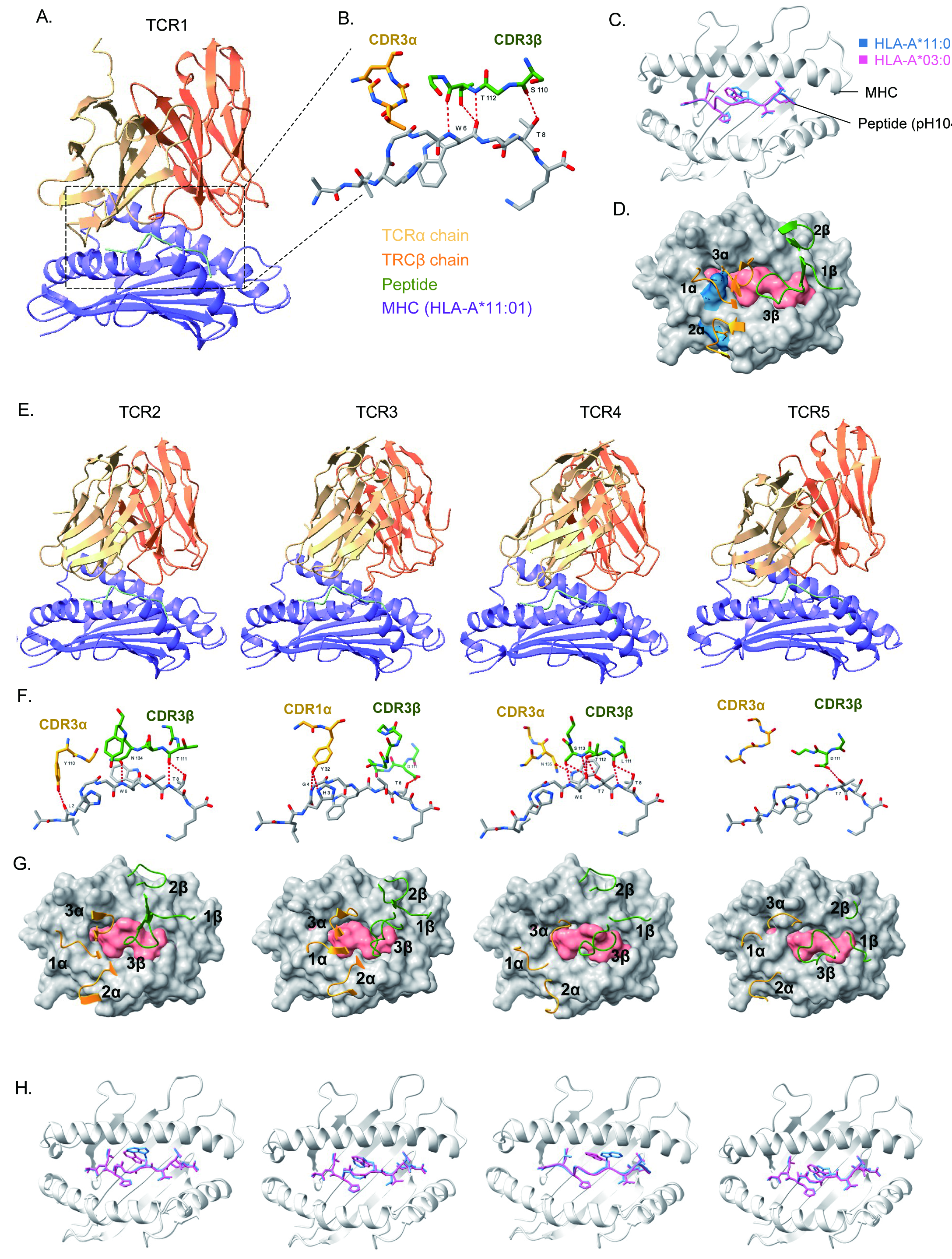

Supplement: Supplementary file 2 — Supplementary Figure 2. (A) Structural overview of the TCR1 pH1047L/HLA-A*11:01 ternary complex. Yellow TCRα chain, orange TCRβ chain, green pH1047L, violent HLA-A*11:01. (B) AAs of TCR1’s CDR3α and CDR3β loops that interact with the pH1047L peptide. Hydrogen bonds are indicated by red. (C) Top view of the conformation of pH1047L peptide presented by HLA-A*11:01 or HLA-A*03:01, when interaction with TCR1. Pink HLA-A*03:01, blue HLA-A*11:01. (D) Top view of the pH1047L/HLA-A*11:01 complex displaying the positions of the six CDR loops of TCR1. Blue AA indicate the different amino acid between HLA-A*03:01 and HLA-A*11:01. (E) Structural overview of the TCR2, 3, 4 and 5 pH1047L/HLA-A*11:01 ternary complex. (F) AAs of TCR2, 3, 4 and 5’s CDR3α and CDR3β loops that interact with the pH1047L peptide. (G) Top view of the pH1047L/HLA-A*11:01 complex displaying the positions of the six CDR loops of TCR2, 3, 4 and 5. (H) Top view of the conformation of pH1047L peptide presented by HLA-A*11:01 or HLA-A*03:01, when interaction with TCR2, 3, 4 and 5. Pink HLA-A*03:01, blue HLA-A*11:01. (TIF 32148 KB) [file 262_2024_3729_MOESM2_ESM.tif]

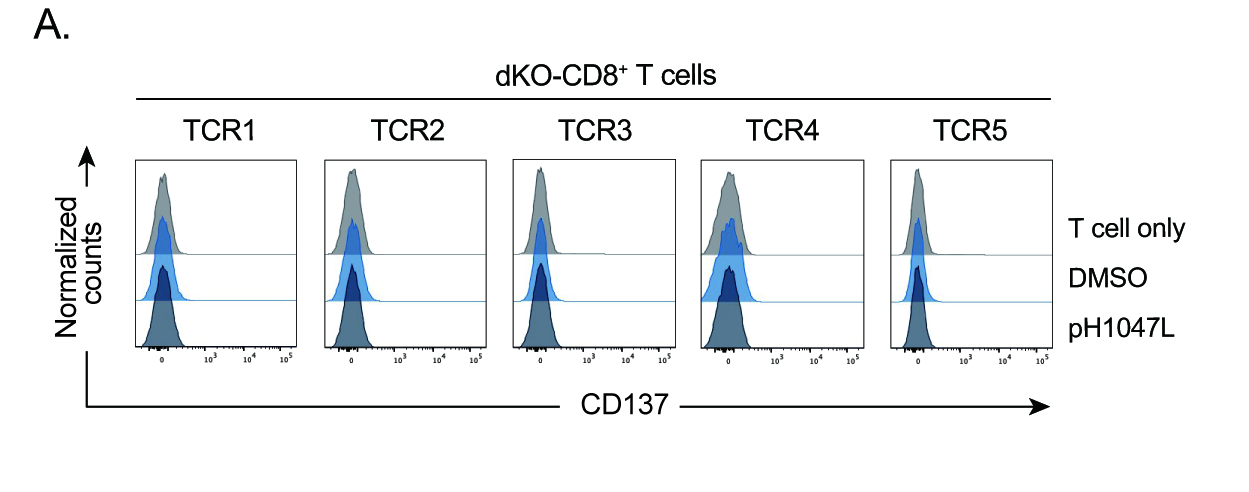

Supplement: Supplementary file 3 — Supplementary Figure 3. (A) Flow cytometric analysis of the percentage of CD137+ pH1047L-specific TCR-T cells after co-cultured with HLA-A*03:01+ K562 cells pulsed with mutant peptide. DMSO was used as control. (TIF 3214 KB) [file 262_2024_3729_MOESM3_ESM.tif]

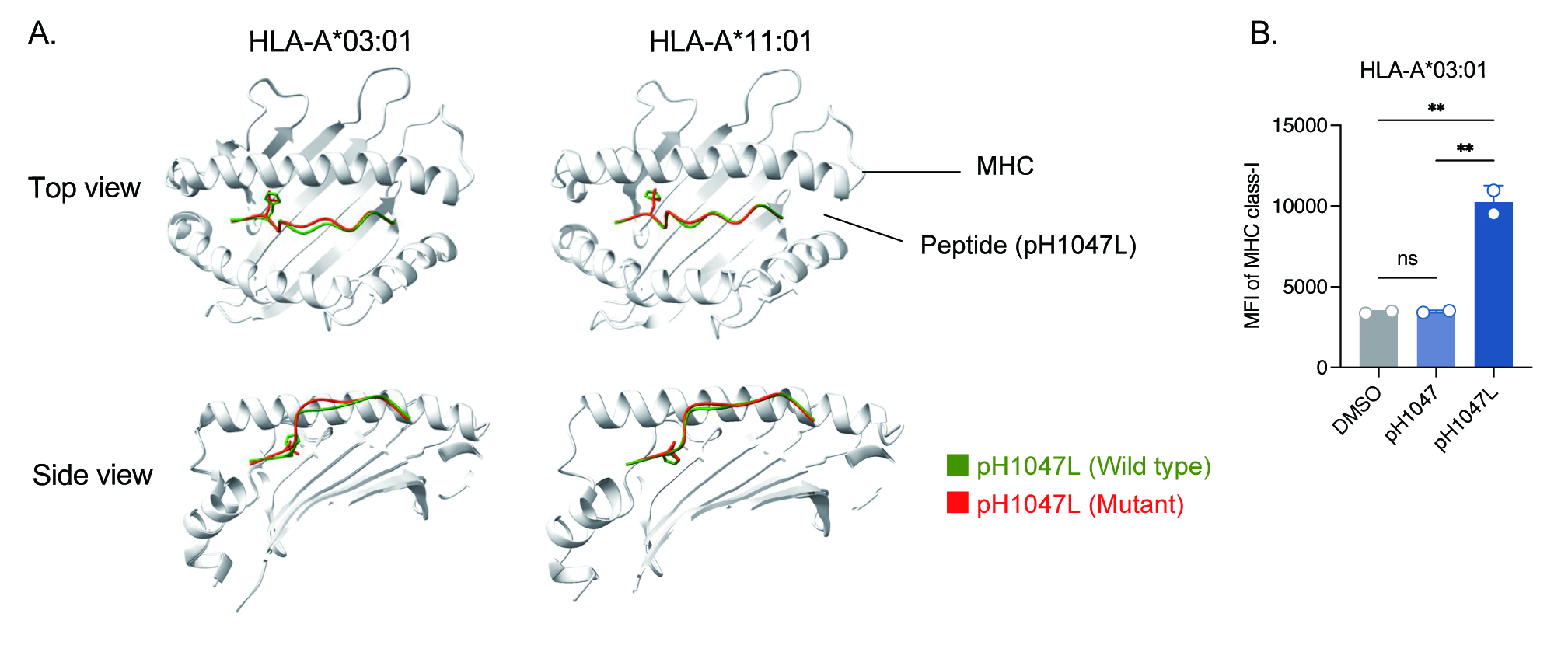

Supplement: Supplementary file 4 — Supplementary Figure 4. (A) Structural superimposition of the pH1047L and pH1047 peptides bound to HLA-A*03:01 or HLA-A*11:01. (B) Stabilization analysis of HLA-A on TAP1-deficient K562 cells expressing HLA-A*03:01. Differences were tested by student’s t-test. **: p < 0.01. ns: denotes not significant. (TIF 8083 KB) [file 262_2024_3729_MOESM4_ESM.tif]
